# Advanced Photocatalysts Based on Conducting Polymer/Metal Oxide Composites for Environmental Applications

**DOI:** 10.3390/polym13183031

**Published:** 2021-09-08

**Authors:** Vinh Van Tran, Truong Thi Vu Nu, Hong-Ryun Jung, Mincheol Chang

**Affiliations:** 1 Alan G. MacDiarmid Energy Research Institute, Chonnam National University, Gwangju 61186, Korea; vanvinhkhmtk30@gmail.com; 2 Advanced Institute of Science and Technology, University of Danang, Danang 50000, Vietnam; Truongnu2@gmail.com; 3 Industry-University Cooperation Foundation, Chonnam National University, Gwangju 61186, Korea; 4 Department of Polymer Engineering, Graduate School, Chonnam National University, Gwangju 61186, Korea; 5 School of Polymer Science and Engineering, Chonnam National University, Gwangju 61186, Korea

**Keywords:** conducting polymer, metal oxides, binary composite, ternary composite, photocatalyst

## Abstract

Photocatalysts provide a sustainable method of treating organic pollutants in wastewater and converting greenhouse gases. Many studies have been published on this topic in recent years, which signifies the great interest and attention that this topic inspires in the community, as well as in scientists. Composite photocatalysts based on conducting polymers and metal oxides have emerged as novel and promising photoactive materials. It has been demonstrated that conducting polymers can substantially improve the photocatalytic efficiency of metal oxides owing to their superior photocatalytic activities, high conductivities, and unique electrochemical and optical properties. Consequently, conducting polymer/metal oxide composites exhibit a high photoresponse and possess a higher surface area allowing for visible light absorption, low recombination of charge carriers, and high photocatalytic performance. Herein, we provide an overview of recent advances in the development of conducting polymer/metal oxide composite photocatalysts for organic pollutant degradation and CO_2_ conversion through photocatalytic processes.

## 1. Introduction

Photocatalysis plays a critical role in the development of emerging technologies for environmental applications, such as wastewater treatment and CO_2_ reduction [1,2,3]. Currently, advanced photocatalytic materials consist mainly of metal oxides, such as TiO_2_, SnO_2_, ZnO, and spinel ferrites [2,4,5]. Among them, TiO_2_ is still the most used semiconductor, comprising about 25% of semiconductors used in the photocatalyst field [6]. Nonetheless, TiO_2_ possesses a wide band-gap energy (~3.2 eV), and, thus, it only absorbs ultraviolet (UV) light, which accounts for only 4% of solar energy [7]. Moreover, TiO_2_ also shows a fast electron–hole recombination rate [4]. These two drawbacks are common challenges in the development of other metal oxides for photocatalytic applications. Therefore, many strategies have been investigated for tailoring and modulating the light adsorption ability, as well as enhancing the charge separation, in metal oxide semiconductors, including (i) band-gap engineering approaches [8]; (ii) developing co-photocatalysts [9]; (iii) doping with metal nanoparticles (i.e., Au, Pt, Cu) [10]; and (iv) design of different composite photocatalysts [11]. Among them, the development of composite photocatalysts has been regarded as one of the most promising approaches due to several advantages. First, the surface properties of metal oxide-based composite structures can be tuned to achieve mid-band-gap electronic states and produce a high absorption in the visible light spectrum [12]. Metal oxide composites can also enhance photocatalyst stability and enable the fabrication of advanced photocatalysts with novel structures, such as microporous, hollow shells, or hierarchical structures.

Conducting polymers have been commonly used as electrocatalysts and photocatalysts as promising alternatives to traditional inorganic semiconductors in various applications (i.e., energy storage, sensors, and environmental protection) because of their superior photocatalytic activities, good conductivities, and unique electrochemical and optical properties [13,14,15]. Techniques for the preparation of conducting polymers are also simple and easy to scale up for large-scale production by using chemical or electrochemical approaches. In addition, conducting polymers with narrow bandgaps enable the absorption of visible light from the sun [14]. Polyaniline (PANI), poly (3,4-ethylenedioxythiophene) (PEDOT), polypyrrole (PPy), and their derivatives are common conducting polymers used in photocatalytic applications for wastewater treatment and CO_2_ reduction. However, there are some problems that severely limit the practical applications of conducting polymers. Most conducting polymers exhibit a low mechanical strength, high brittleness and poor processability [16]. In order to overcome these limitations, the fabrication and development of organic–inorganic composite photocatalysts based on organic conducting polymers and metal oxides has been considered as a promising approach for environmental applications [17]. In these composites, conducting polymers play a role as the supporting matrix for the intercalation of catalytically metal oxide nanoparticles and photosensitizers for the enhancement of light adsorption in the visible spectrum, which improves the photocatalytic performance and stability of the composite photocatalysts.

To date, many studies on the synthesis and development of novel conducting polymer/metal oxide composites have been reported. However, there has not yet been a review article that provides a systematic overview of the reported studies on these composites. Therefore, this review aims to summarize recent advancements in photocatalytic conducting polymer/metal oxide composites for environmental applications. We also explain and discuss the photocatalytic mechanisms and outline some problems related to the use of these composites in practical applications.

## 2. Synthesis and Properties of Conjugated Polymer/Metal Oxide Composites

Composites of conjugated polymers (CPs) and transitional metal oxides exhibit a significant improvement in photocatalytic performance [14,18,19]. The hybrid material possesses a high photoresponse and delayed recombination of charge carriers due to distinct exciton-plasmon interactions and a higher surface area for light absorption. Consequently, composites of CPs and wide band-gap inorganic semiconductors, especially metal oxides, have been attracting attention for their potential use in photocatalytic and photoelectric conversion applications. It has been reported that CP/metal oxide composites often act as p-n junction semiconductors that can be tailored by combining a p-type CP with an n-type metal oxide semiconductor in order to overcome the problems related to a high rate of electron–hole recombinations, poor activation with visible light, leaching and thermal decomposition. In combination with metal oxides, PANI, PEDOT, and PPy are widely used to prepare composite photocatalysts for the photocatalysis processes needed in environmental applications and CO_2_ reduction, as summarized in Table 1 and discussed in following sections.

In general, there are two common types of the CP/metal oxide composite-based photocatalysts: (i) binary composite and (ii) ternary composite. Binary composites contain one transition metal oxide and one CP [21]. Ternary composites are made of one binary metal oxide composite (e.g., TiO_2_/Fe_3_O_4_ and Cu_2_O/ZnO) and one CP or one metal oxide and one binary composite of CP with another material, such as carbon nanotube and graphene [39].

### 2.1. Binary Composites of CPs and Metal Oxides

#### 2.1.1. PANI/Metal Oxide Composites

Polyaniline (PANI) is one of the most common CPs in photocatalytic applications due to its high stability, high processability, and tunability of the conducting and optical properties [42]. Generally, PANI has a prolonged alternate σ and π bond electronic cloud system, resulting in a significant energy band gap of 2.8 eV [43]. When irradiated by UV–Vis and ultraviolet light, PANI can work as an extraordinary e and h (donor-acceptor) photosensitizer [44]. PANI can be blended with a wide range of metal oxides, such as TiO_2_, ZnO, Fe_3_O_4_, SnO_2_, Sn_3_O_4_, and spinel ferrites to prepare composite photocatalysts.

*PANI/TiO_2_ composites*: Due to a wideband gap, TiO_2_ primarily absorbs UV-light, which accounts for only about 3–5% of sunlight, and limits its photocatalytic ability in the visible region [45,46]. However, PANI exhibits high absorption coefficients in the visible light range, charge carriers with a high mobility, non-toxicity, and low-cost synthesis, and in addition is an excellent electron donor [47]. As a result, PANI is often used to delay the recombination of electron–hole pairs, enhance the charge separation efficiency, and improve the photocatalysis efficiency of TiO_2_ [21,22]. Generally, PANI/TiO_2_ composites are often fabricated through an in-situ chemical oxidation process [48]. The photocatalytic activity of PANI/TiO_2_ can be significantly reduced due to aggregations caused by TiO_2_ particle collisions. However, the aniline molecules tend to create a barrier to the aggregation processes of TiO_2_ nanoparticles and PANI protects the TiO_2_ surface from blockage by intermediates. It has been supposed that the synergetic effect between PANI and TiO_2_ increases the photocatalytic activity of the obtained composites. It has been reported that there are different TiO_2_ nanostructures that can be incorporated with PANI, such as nanorods, mesoporous, nanobelts, and nanotubes [20,21,22,23]. The band-gap energy of PANI/TiO_2_ composites can be reduced to 2.77–3.1 eV.

*PANI/ZnO composite*: Like TiO_2_, ZnO is also a widely investigated semiconductor due to its abundance, low cost, and low toxicity [49]. Nonetheless, the recombination of photogenerated charge carriers remains a dominant barrier which significantly limits the practical applications of ZnO, especially on a large scale. Defect-related mediation has been considered as a potential approach in order to modulate the activity of ZnO, and defects on the outer surface of ZnO which can boost its photocatalytic activity remarkably in degradation and photoreduction reactions [50,51]. Pei et al. successfully developed a composite of PANI and defect-rich ZnO using the chemisorption method [52]. The monomolecular-layered PANI can stabilize the surface of ZnO. Additionally, it is expected that a surface of ZnO coated by PANI molecules results in a PANI-based ZnO catalyst with higher photocatalytic activity, while preventing the photo-corrosion of ZnO, even for the monomolecular form of PANI [24]. Under visible-light irradiation, PANI molecules generate a π-π* transition and deliver the excited electrons to the conduction band (CB) of ZnO, which reduces the band-gap energy of ZnO [25]. The PANI/ZnO composite photocatalysts are expected to be promising candidates for the design of high-activity, high-stability, and visible-light-driven photocatalysts in the future.

**Figure 1:**
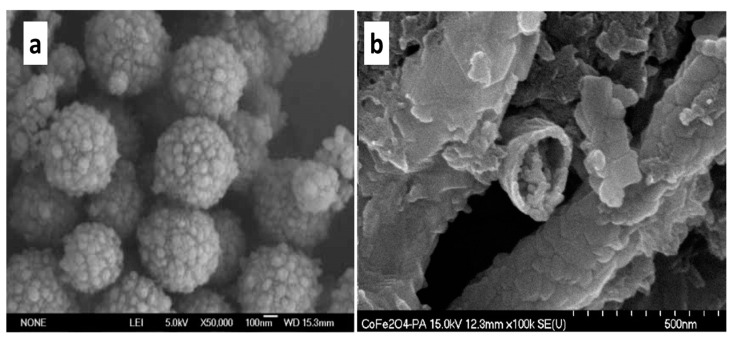
(**a**) SEM image of the as-prepared Fe_3_O_4_/PANI core/shell composite [54]. (**b**) CoFe_2_O_4_/PANI hollow nanofibers [29].

*PANI/magnetic iron oxide composites*: PANI and magnetic iron oxide hybrid materials have been demonstrated to be good candidates for the photodegradation of organic dyes in wastewater, due to their electrical conductivity properties associated with superparamagnetism [26]. For example, Alves et al. prepared a composite of PANI and magnetic iron oxide by in-situ chemical polymerization for the adsorption/photodegradation of blue methylene dye. In this photocatalyst, iron oxide nanoparticles (10–15 nm) were embedded in/on the polymer matrix and the synergistic effects of the iron oxide particles and polymer phases were considered to be responsible for the photocatalytic action and the high absorption behavior. In another study, Yang et al. also developed a composite of PANI and Fe_3_O_4_ nanoparticles, in which the polymerization of aniline was catalyzed by Fe_3_O_4_ nanoparticles [53]. In general, PANI/Fe_3_O_4_ composites often have a core/shell structure (Figure 1a) [54].

*PANI/Tin oxide composites:* SnO_2_ is considered to be a potential alternative to TiO_2_ owing to being a better electron acceptor and having a more positive CB potential [55]. However, SnO_2_ has showed low efficiency in the utilization of solar energy and low photocatalytic efficiency because of the fast recombination of its photogenerated electrons and holes [56,57]. It has been indicated that PANI has a matching electronic band structure with SnO_2_ and that they can be combined to obtain a type-II SnO_2_/PANI heterojunction. In this composite, PANI transfers its photogenerated electrons to the CB of SnO_2_; thus, it plays an important role as a photosensitizer for SnO_2_ under visible light [58]. Moreover, the metal oxide and the hydroxyl group of SnO_2_ can be altered slightly with the substitution of PANI. Compared with bare SnO_2_ nanoparticles, the crystallite size of SnO_2_ in the composite can be significantly decreased, while its surface area was increased due to the inclusion of PANI [27] Consequently, the substitution of PANI reduced the reflectance and band-gap energy (3.1–2.7 eV) of SnO_2_, resulting in the SnO_2_/PANI composite working effectively in the visible light range [59]. In addition to common PANI/SnO_2_ composites, Manfei et al. recently reported the coupling of a p-type PANI with an n-type Sn_3_O_4_ for photocatalytic applications [28]. In that study, a Sn_3_O_4_ nanosheet composite was modified by PANI nanofibers and a p–n PANI/Sn_3_O_4_ heterojunction was successfully prepared via a mechanical milling method. The photocatalytic activity and stability of the PANI/Sn_3_O_4_ composite were highly improved compared with single Sn_3_O_4_.

*PANI/spinel ferrite composites:* Spinel ferrite nanoparticles (SFNPs) are defined as metal oxides with the spinel structure, with the general formula of MFe_2_O_4_, where M = divalent cations [60]. The physicochemical properties of SFNPs depend mainly on the types, amounts, and positions of the M cations in the crystallographic structure. MFe_2_O_4_ nanoparticles exhibit some advantages, such as stability, biocompatibility, low-cost, excellent magnetic properties, and easy separation [61]. Therefore, they have been widely used in the development of binary nanocomposites as photocatalysts for the photodegradation of pollutants. Several SFNPs, including CoFe_2_O_4_, CuFe_2_O_4_, MnFe_2_O_4_, NiFe_2_O_4_ and ZnFe_2_O_4_ have been reported as potential candidates for the development of composite photocatalysts with CPs [62,63,64]. Combinations of MFe_2_O_4_ with different concentrations of CPs have been demonstrated to be an effective approach for improving the photocatalytic performance, with PANI being especially effective. For example, Kim et al. fabricated CoFe_2_O_4_/PANI hollow core-double shell nanostructures as a composite photocatalyst using the electrospinning technique and in-situ chemical oxidative polymerization (Figure 1b) [29]. The results showed that owing to the heterojunction built between CoFe_2_O_4_ and PANI, the hollow CoFe_2_O_4_/PANI composite easily captured visible light and exhibited effective charge separation, which resulted in a significant improvement in visible light photocatalysis.

#### 2.1.2. Composites of PEDOT and Metal Oxides

PEDOT is known as a conducting polymer that exhibits a narrow band gap (E = 1.69 eV) and an excellent ability to absorb light in the visible and near infrared regions [65]. PEDOT based photocatalysts exhibit good stability, good recyclability, and reusability [30]. Therefore, PEDOT has been commonly coupled with a wide range of metal oxides as composite photocatalysts. Among them, TiO_2_ and ZnO are often combined with PEDOT in the preparation of composites for photocatalytic applications.

**Figure 2:**
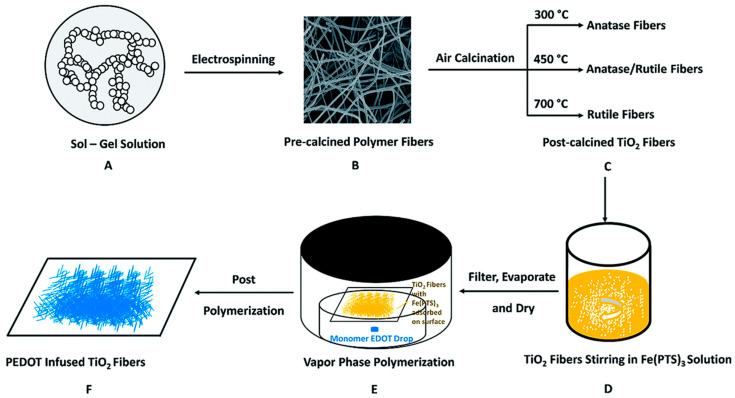
The preparation process of PEDOT infused TiO_2_ nanofibers via electrospinning and calcination [67]: (**A**) Sol–gel solution containing PMMA, precursor TTIP and the solvents; (**B**) post-electrospinned polymer fibers containing PMMA, amorphous TiO_2_ and solvent without evaporating; (**C**) post-calcined TiO_2_ nanofibers under different calcination temperatures with different phase compositions; (**D**) stirring of post-calcined TiO_2_ nanofibers with yellow oxidant Fe(PTS)3 solution; (**E**) dried TiO_2_ nanofibers with adsorbed Fe(PTS)3 on surface were put in a heated chamber for VPP reaction; (**F**) bluish dried PEDOT infused TiO_2_ nanofibers on filter paper.

*PEDOT/TiO_2_ composite:* PEDOT is regarded as an attractive CP for coupling with TiO_2_ in composite materials for visible-light-driven photocatalytic applications. Similar to PANI, it can be photoexcited under visible-light irradiation to transfer electrons into the CB of TiO_2_, which leads to effectively separate holes (h^+^) and electrons (e^−^) and increases the number of photoexcited charges available to drive photoreactions substantially [30]. The PEDOT/TiO_2_ composite was developed to overcome disadvantages related to the lower photon transport of the TiO_2_ surface by incorporation into the polymer layer. Generally, the PEDOT coating thickness is considered to be an important parameter during the fabrication process and must be controlled, so that photogenerated charge carriers can be easily transported from the external polymer interface to the inner TiO_2_ layer [66]. Recently, Liu et al. demonstrated the enhanced photocatalytic performance of PEDOT to TiO_2_ nanofibers by improving the rate of transformation of photogenerated holes (Figure 2) [67]. The PEDOT/TiO_2_ nanofiber composite was fabricated via electrospinning and calcination to form TiO_2_, followed by the introduction of PEDOT using vapor phase polymerization.

*PEDOT/ZnO composite*: PEDOT was used to increase the photocatalytic activity of ZnO due to its efficient electron donor and good electron transporters upon visible-light irradiation. For instance, Abdiryim et al. introduced a simple solid-state heating method to prepare PEDOT/ZnO nanocomposites in powder form with the content of ZnO varying between 10 and 20 wt% [68]. The photocatalytic activity of these nanocomposites can be enhanced by the incorporation of ZnO nanoparticles under both UV and visible-light irradiation, which can be ascribed to the high charge separation of electron and hole pairs in the obtained composite.

#### 2.1.3. Composites of PPy and Metal Oxides

Owing to superior conductivity, high charge carrier mobility, high absorption coefficient in the visible light, and good environmental stability, polypyrrole (PPy) is one of the most promising candidates for the development of stable photosensitizers to improve the photocatalytic activity and solar light conversion efficiency of metal oxides [69].

*PPy/TiO_2_ composite:* PPy/TiO_2_ nanocomposites have been mainly applied in the photocatalytic degradation of organic species. Currently, there are numerous methods that can be used to synthesize PPy/TiO_2_ nanocomposites, such as anodic co-deposition [70], self-assembly techniques [71], photo-electrochemical polymerization [72], and hydrothermal methods [31]. For practical environmental applications, however, the in-situ chemical oxidation method has been considered as the most promising technique due to its simplicity, good reproducibility, and possibility for large-scale production [32]. For instance, Gao et al. successfully prepared PPy/TiO_2_ nanocomposites using a facile chemical oxidation of pyrrole in a prepared TiO_2_ sol solution [32]. For this composite, a PPy film of about 2–3 nm was coated onto the TiO_2_ surface, which is supposed to increase the photocatalytic activity for the degradation of rhodamine B and the reduction of CO_2_. Moreover, PPy nanostructures (i.e., nanofibers and nanospheres), can be synthesized by directly oxidizing pyrrole (Py) monomers in a solution under mild oxidation conditions and a low temperature [73]. Based on this approach, Dimitrijevic et al. developed a simple one-step hydrothermal method for fabricating PPy/TiO_2_ nanocomposites [31]. According to this method, 4.5 nm TiO_2_ nanoparticles were electronically coupled to 200−300 nm PPy granules to form a stable composite, which is capable of efficient visible-light photocatalysis. In this composite, PPy molecules act as visible-light photosensitizers, and the photocatalytic activity of the composite increases through the enhanced electron transfer from excited PPy to TiO_2_ nanoparticles. Importantly, it has been shown that a high concentration of TiO_2_ nanoparticles used in the composite can significantly increase the photocatalytic efficiency of the PPy/TiO_2_ composite.

**Figure 3:**
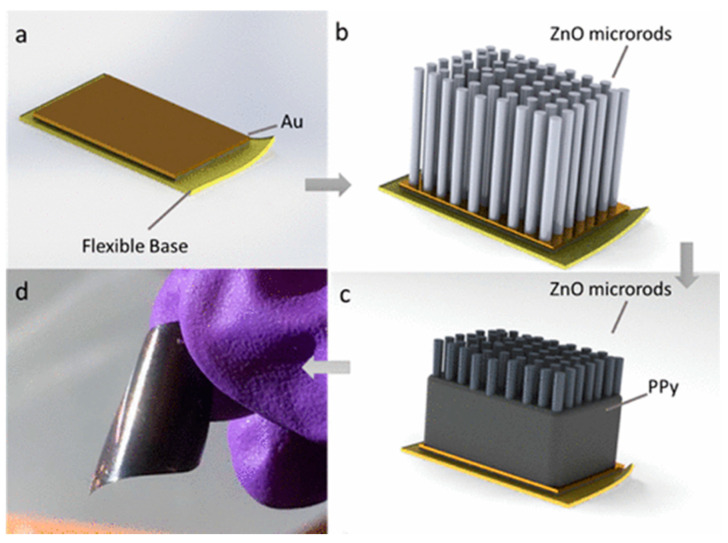
Schematic illustration of the preparation process of a flexible ZnO-microrod/PPy composite film. (**a**) Flexible base polyester film. (**b**) ZnO-microrod arrays were grown on the base polyester film. (**c**) PPy was electrodeposited onto the ZnO-microrod arrays. (**d**) Optical image of highly flexible ZnO-microrod/PPy composite film (20 mm × 20 mm) [33].

*PPy/ZnO composite:* With regards to photocatalytic activity, PPy donates photon-induced electrons to ZnO under visible-light irradiation, which results in an improvement in the photocatalytic activity and a reduction in the recombination of charge carriers [74,75]. To create a flexible photocatalytic film, a novel composite of ZnO-microrod arrays and electrodeposited PPy was recently developed (Figure 3) [33]. To prepare the composite film, the upper section of a ZnO microrod is first covered with a very thin PPy shell, while the lower section of the ZnO microrod is coated with a thick PPy base layer. In this composite, the upper PPy shell works as a photosensitizer through the absorption of visible light and then, the conversion of photons into free carriers (i.e., electrons and holes) [76,77,78], whereas the lower PPy base layer will stabilize the ZnO-microrods on the flexible substrate and facilitate the electron transport to the substrate [79]. It has been suggested that the accelerated carrier separation at the ZnO/PPy interface leads to a considerable enhancement in the photocatalytic activity of ZnO/PPy composite films [80]. Moreover, due to the unique structure integrating flexibility, sunlight-driven photocatalytic properties, and high mechanical strength, ZnO/PPy composite films show a high potential for use in flexible electronics and other applications in the environmental field.

### 2.2. Ternary Composites of CP/Metal Oxides

Based on the above section, it can be concluded that binary composites of CPs and metal oxides can significantly enhance the photocatalytic activity of individual semiconductors in the visible light region. However, recovery and reusability of photocatalysts are also considered as important factors for practical applications. Recently, ternary composites based on CPs and metal oxides have led to new insights into the design and development of novel multicomponent photocatalysts with versatile and extraordinary properties [37,39,41,81,82]. Therefore, the preparation and design of multicomponent nanocomposites for further improving catalytic performances is of great interest. It has been demonstrated that the formation of a Z-scheme heterojunction can effectively improve carrier mobility, while the synergetic interactions of the components could also maintain the redox ability of the generated electrons and holes for a very long time [83]. Currently, the preparation of ternary nanocomposites based on CPs and metal oxides for creating Z-scheme heterojunctions has attracted further attention for improvements in the properties of metal oxides and binary composite photocatalysts due to two superior advantages: (i) inhibition of corrosion and stabilization of CPs; (ii) synergistic enhancement of the three components [37]. It is strongly believed that a ternary nanocomposite of CP, metal oxide, and another compound will show an enhanced photocatalytic activity in terms of a low band-gap energy, minimized recombination rate and strong absorption of visible light due to the synergism effect between the constituents [39].

As mentioned earlier, the combination of PANI and TiO_2_ can enhance the photo activity of TiO_2_ into the visible light region. Moreover, incorporating magnetic nanoparticles into the binary composites of PANI/TiO_2_ ensures that the photocatalyst can easily handle magnetic separation [84] and provides an effective approach for achieving a more efficient charge separation, resulting in increased photocatalytic activity [85,86]. Taking all these points into consideration, Xiong et al. prepared a magnetically recyclable ternary TiO_2_-CoFe_2_O_4_-PANI composite photocatalyst using in-situ oxidative polymerization [34]. The results indicate that ternary TiO_2_-CoFe_2_O_4_-PANI composites show highly enhanced photocatalytic activity for the visible light region compared with binary TiO_2_-CoFe_2_O_4_, CoFe_2_O_4_-PANI, or TiO_2_-PANI composites. Additionally, this ternary composite photocatalyst can be easily separated out and reused by simply using an external magnetic field after the reaction, owing to the good magnetic properties of CoFe_2_O_4_ nanoparticles. Using a similar approach, Li et al. also confirmed the enhanced electrical conductivities and photocatalytic activity of ternary ZnFe_2_O_4_-TiO_2_-PANI composites with different amounts of PANI [35]. Moreover, it has been indicated that interactions between individual components in these ternary composites result in an enhancement of their electrical conductivities and photocatalytic activities, which is an especially important purpose of PANI coating. As the mass fraction of aniline was up to 50%, the ternary composite exhibited considerable photocatalytic activity and displayed excellent reusability.

**Figure 4:**
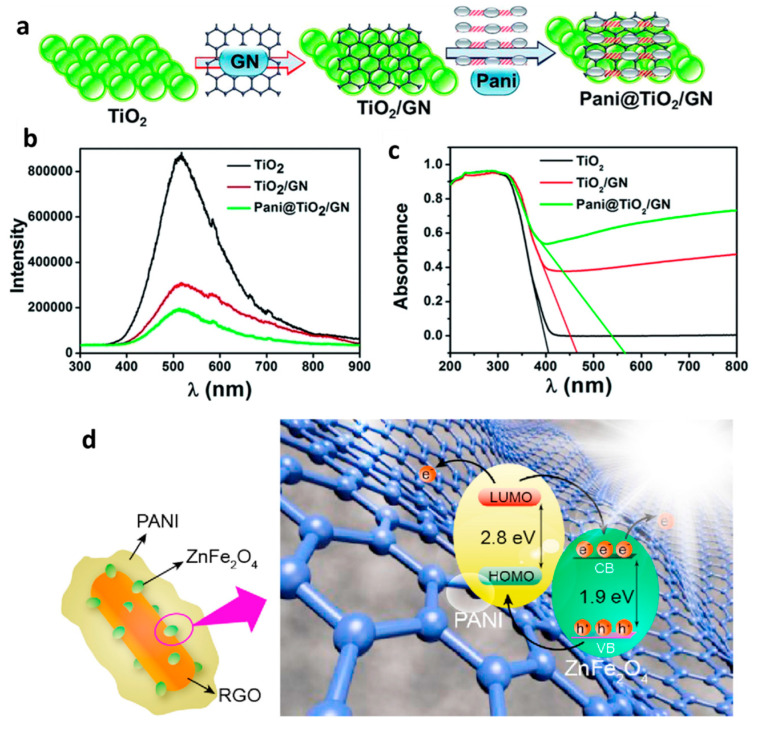
(**a**) Schematic representation of the synthesis of PANI/TiO_2_/graphene nanocomposite (**b**) PL and (**c**) UV–Vis diffuse absorbance spectra of TiO_2_, TiO_2_/GN and PANI/TiO_2_/graphene nanocomposite [39]. (**d**) Microstructure and photocatalytic mechanism diagram of ternary GO-ZnFe_2_O_4_-PANI composite [36].

Graphene, which is well-known as a 2D layered hexagonal lattice of carbon nanomaterials [87], is a potential material for increasing the photocatalytic efficiency and stability of composite photocatalysts, due to its superior electronic and transport properties, high surface area, and zero band-gap energy [88]. In addition, it has been demonstrated that graphene can accept electrons and therefore inhibit recombination and increase the absorption properties and stability of composite catalysts [89,90,91]. Therefore, nanocomposites of various semiconductors with graphene (GN) have been investigated as advanced photocatalysts [92,93,94]. It has been reported that the fabrication of ternary composites based on graphene and reduced graphene oxide (rGO) with transition metal oxides and conducting polymers can be considered to be a promising approach for overcoming the problems related to recombination losses and developing novel photocatalysts with perfect photocatalytic activity [36,95,96,97]. According to this methodology, there is a diversity in ternary composites in combination with graphene or graphene oxide, PANI, and metal oxides that have been recently introduced in the degradation and absorption of organic pollutants. For example, Kumar et al. successfully developed a conducting ternary PANI/TiO_2_/graphene nanocomposite through an in-situ oxidative polymerization method, in which aniline molecules were polymerized in the presence of TiO_2_ and graphene nanoparticles (Figure 4a) [39]. UV–Vis absorption and PL spectra showed that PANI/TiO_2_/graphene exhibited a higher visible light absorption and a lower recombination rate than PANI/TiO_2_ (Figure 4b,c). Using a combination of rGO, PANI, and spinel ferrite, Feng et al. synthesized a rGO/ZnFe_2_O_4_/PANI ternary photocatalyst via a simple and low-cost method (Figure 4d) [36]. In the first step, spinel ZnFe_2_O_4_ nanoparticles were deposited onto the surface of rGO to form the binary rGO/ZnFe_2_O_4_ composite. This binary composite was then coated with PANI to obtain the ternary composite in the next step. It was shown that this ternary composite structure exhibited three main advantages: (i) all spinel ZnFe_2_O_4_ nanoparticles in the binary rGO/ZnFe_2_O_4_ composite were completely coated with PANI, and thus the photoinduced e^−^-h^+^ pairs produced by ZnFe_2_O_4_ could be stabilized by PANI; (ii) the photoinduced e^−^-h^+^ pairs produced by PANI were also stabilized by rGO; (iii) the synergistic effect between the three components significantly enhanced the photocatalytic activity of the ternary composite photocatalyst. In another study, Miao et al. reported a ternary hybrid through a combination of rGO and a binary PANI/Cu_2_O composite via a one-pot method [97]. In this composite, PANI and Cu_2_O nanoparticles were embedded in the rGO nanosheets, which highly increased the photoactivities of ternary nanocomposites compared with the binary ones of PANI/Cu_2_O, rGO/Cu_2_O, or PANI/rGO. In summary, it is expected that the preparation of ternary photocatalysts based on graphene or rGO, and PANI with metal oxides will be a new and promising pathway for the development of advanced photocatalysts.

Recently, the combination of TiO_2_, graphitic carbon nitride (g-C_3_N_4_), and PANI has also been investigated in an attempt to discover novel ternary composite photocatalysts by taking advantage of each component [98]. These composites are expected to have a higher interfacial charge transfer, which can greatly enhance the photodecomposition of organic pollutants. For example, Alenizi et al. reported a ternary g-C_3_N_4_/TiO_2_/PANI nanocomposite photocatalyst for the degradation of organic dyes in wastewater [41]. Regarding the synthesis process, a defect-rich TiO_2_ lattice and lamellar structures were first generated from TiO_2_ powders mixed with 10 M NaOH. It was indicated that mixed phase titania and sodium titanate lamellar structures resulted in a better surface area for binding with g-C_3_N_4_ and PANI, which promoted the higher interfacial charge separation and improved the adsorption-photocatalytic properties. Moreover, the g-C_3_N_4_/TiO_2_/PANI nanocomposite showed a higher photocatalytic activity under direct sunlight irradiation. In another study, such a novel ternary heterostructure also showed a considerable increase in charge separation efficiency, specific surface area and visible light harvesting, which can be attributed to the synergetic effects of PANI and ZnO and the exfoliated two dimensional CN nanosheets in the roles of catalysts and supporting materials, respectively [98].

## 3. Visible-Light-Responsive Photocatalysis Mechanisms of Conducting Polymer/Metal Oxide Composites

In terms of the general mechanism, a semiconductor photocatalyst enables the absorption of visible light from the solar spectrum, which causes the excitation of electrons from the valence band (VB) to the CB and generates electron–hole pairs. These electrons and holes are then transferred to the composite photocatalyst surface for the degradation and oxidization of CO_2_ or pollutants.

**Figure 5:**
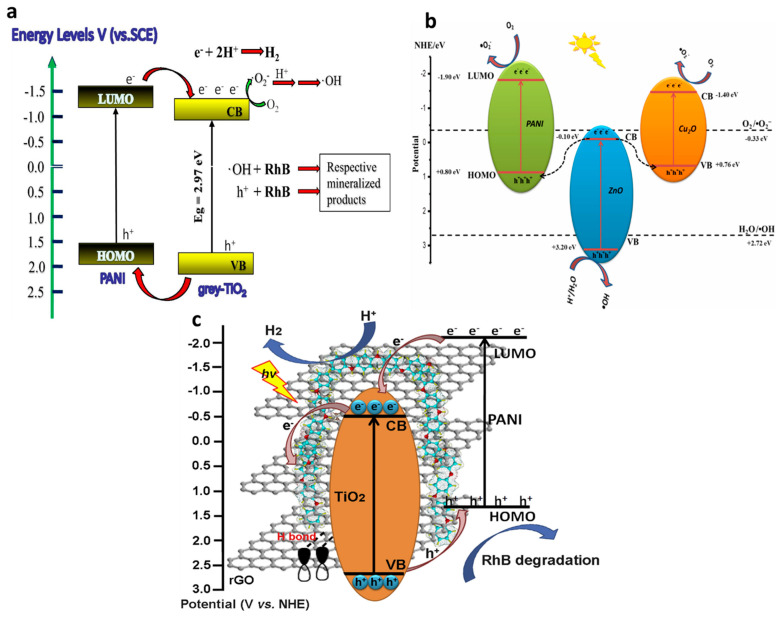
(**a**) Proposed mechanism for the photocatalytic degradation using a binary PANI/TiO_2_ composite under visible-light irradiation [101]. (**b**) Schematic diagram of the charge transfer pathway in a ternary Z-scheme Cu_2_O/ZnO-PANI composite under visible-light irradiation [38]. (**c**) Mechanism of photocatalytic activity of ternary PANI/TiO_2_/rGO composite [102].

In the binary composites of conducting polymers and metal oxides, conducting polymers work as visible photosensitizers to generate photoelectrons from the VB to the CB, which can be transferred to the CB of the metal oxides [99]. This is especially the case in relation to the PANI/TiO_2_ composite, and the processes of photoexcitation, charge separation and reaction in the composite under visible-light irradiation is presented in Figure 5a [100,101]. It has been demonstrated that the energy levels of TiO_2_ and PANI can be matched to each other [23]. The CB of TiO_2_ is slightly lower than the LUMO of PANI, and thus TiO_2_ can work as a sink for the photogenerated electrons in the composite photocatalyst. Furthermore, the HOMO of PANI is higher than the VB of TiO_2_, and thus PANI can act as an acceptor for the photogenerated holes in the composite photocatalyst. Consequently, the adsorption and electrical conductivity of the binary PANI–TiO_2_ composite under visible-light irradiation is significantly boosted, and larger numbers of electron–hole pairs are generated. Specially, under visible-light irradiation, PANI will absorb photons to induce electrons into the LUMO, while TiO_2_ will absorb the UV–Vis light to excite the electrons into the CB. Due to the different potentials of PANI and TiO_2_, as mentioned previously, the excited electrons in the LUMO of PANI can be transferred to the CB of TiO_2_ and the generated h^+^ can move from the VB of TiO_2_ to the HOMO of PANI. The photoelectrons on the surface of the composite will reduce H^+^ to form H_2_ or react with the surface-adsorbed O_2_ to generate ●OH radicals, which play a main role in the degradation of pollutants. Meanwhile, photo-holes enable pollutants to oxidize mineralized products.

For ternary composites of conducting polymers and metal oxides, the mechanism can be explained based on two different types of composites: (i) conducting polymer/metal oxide/metal oxide; (ii) conducting polymer/metal oxide/another compound. Regarding the conducting polymer/metal oxide/metal oxide composites, it has been indicated that the formation of Z-scheme composites is likely to effectively enhance the carrier mobility and generation of electrons and holes for a very long time [83]. In these Z-scheme composite photocatalysts, photogenerated electrons from a metal oxide recombine with the photogenerated holes in another coupled metal oxide [99,103]. For example, the ternary composite Cu_2_O/ZnO- PANI showed Z-scheme heterojunction properties, which resulted in super-fast photocatalytic activities and high stability [38]. The photocatalytic mechanism of the Z-scheme Cu_2_O/ZnO- PANI composite is presented in Figure 5b. It is obvious that the photogenerated electrons in the CB of ZnO are rapidly transferred to the VB of Cu_2_O and the HOMO of PANI, where they recombine with the photogenerated holes. At the same time, the photogenerated electrons in the LUMO of PANI and the CB of Cu_2_O are separated and migrate to the surface to react with surface-adsorbed O_2_ in order to generate ●O_2_^−^ radicals, while the photogenerated holes in the VB of ZnO are also transferred to the surface for the photocatalytic evolution of ●OH radicals. These formed radials are mainly responsible for the photodegradation and photoreduction of pollutants.

Regarding ternary composites of conducting polymer/metal oxide/another compound, the cooperation between the three components is expected to improve the photoinduced charge separation and suppress charge recombination, resulting in an enhanced photocatalytic performance. For instance, the highly enhanced photocatalytic activity of a ternary PANI/TiO_2_/rGO composite is significantly contributed to by the interfacial charge transfer in PANI and rGO due to p-conjugated groups [102]. This detailed photocatalytic mechanism is shown in Figure 5c. In this composite, the ●O_2_^−^ radicals for the oxidization and degradation of the pollutants are generated by similar pathways as in a binary PANI/TiO_2_ composite. Moreover, the hydrogen bonds between TiO_2_ and rGO also promote the migration of photogenerated electrons and holes to the CB of TiO_2_. Moreover, photoinduced electrons can easily be transferred in the composite due to the π-π stacking of rGO, which leads to a long-term maintenance of electron and hole separation, thereby resulting in the significantly enhanced photocatalytic activity of the composite.

## 4. Photocatalytic Applications of CP/Metal Oxide Composites in the Environment Field

Generally, composite photocatalysts are typical semiconductors with an adequate band-gap energy that can strongly absorb light from the solar spectrum, leading to the excitation of electrons from the VB to the CB and, then, the formation of electron–hole pairs. Finally, the electrons and holes move to the photocatalyst surface and react with the absorbed pollutants. As advanced photocatalysts, CP/metal oxide composites can be used in various applications in the environmental field. Four main applications will be discussed in the following section, including the decomposition of organic pollutants, solar water splitting, CO_2_ reduction, and N_2_ reduction.

### 4.1. Decomposition of Organic Pollutants

As mentioned previously, the photocatalytic performances of single components of CPs (PPy, PANI, and PEDOT) and metal oxides (e. g., TiO_2_, ZnO, Fe_3_O_4_, and ZnFe_2_O_4_) are poor. However, their combination can greatly enhance performance due to inhibiting the recombination of charge carriers. Actually, in case of the binary composites of CP and metal oxides, CPs play an important role as photosensitizers in the absorption of visible light, while transition metal ions (Co^2+^, Fe^2+^, Mn^2+^, Ni^+^, etc.) can activate peroxymonosulfate to generate sulfate radicals (SO_4_^•^) and hydroxyl radicals (^•^OH) for the degradation of organic pollutants [104,105]. Most binary composites of CPs and metal oxides have a high photocatalytic activity in the degradation of organic dyes such as bisphenol A, direct blue 15, RhB, methyl orange (MO), methylene blue (MB), and congo red, as presented in Table 1. Taking PANI/SnO_2_ as an example, these binary composites have been commonly used to enhance the photocatalytic degradation of various organic pollutants in wastewater [106,107]. It has been shown that the inclusion of PANI in SnO_2_ results in a decreased crystallite size and an increased surface area. Recently, the photocatalytic efficiencies of SnO_2_/PANI and PANI/Sn_3_O_4_ nanocomposites were evaluated with direct blue 15 [27] and rhodamine B [28]. The results indicate that the PANI/Sn_3_O_4_ composite can reach a maximum degradation efficiency of around 97% for rhodamine B under visible-light irradiation, which is 2.27 times higher than that of Sn_3_O_4_ alone. In addition, the photocatalytic performance of PANI/Sn_3_O_4_ exhibited a relative stability during RhB photodegradation after three runs and the photodegradation efficiency could still be maintained at >90%.

Recently, PANI and magnetic iron oxide composite photocatalysts have been considered as good candidates for the adsorption and photodegradation of organic dyes in the treatment of wastewater due to their high charge transport properties and superparamagnetism at room temperature [108,109]. For instance, Alves et al. reported a PANI/magnetic iron oxide composite for MB removal [26]. Owing to the dispersion of magnetic iron oxide particles in the polymer phase, the composite showed a high electrical conductivity (10^−2^ S·cm^−1^) and good adsorption properties. Interestingly, the PANI/magnetic iron oxide composite showed both adsorption and photodegradation properties in the pollutant removal process. The composite exhibited a reduction efficiency of 99% of the initial dye concentration because of the synergism effect between the iron oxide-polymer phases in the photocatalytic action. Importantly, due to the super-paramagnetic behavior, the PANI/magnetic iron oxide composite can be easily collected by applying a magnetic field and, therefore, can be reused in numerous cycles. Using a similar approach, Kharazi et al. introduced the novel binary composite of copper spinel ferrite (CuFe_2_O_4_) and PANI as an adsorbent and photocatalyst for the treatment of MO in wastewater [108]. With the presence of pre-synthesized CuFe_2_O_4_ nanoparticles, the nanocomposite has a mesoporous structure with a BET surface area of 20.3668 m^2^/g, which showed excellent adsorption of the dye (capacity of 345.9 mg/g) and excellent magnetic properties.

**Figure 6:**
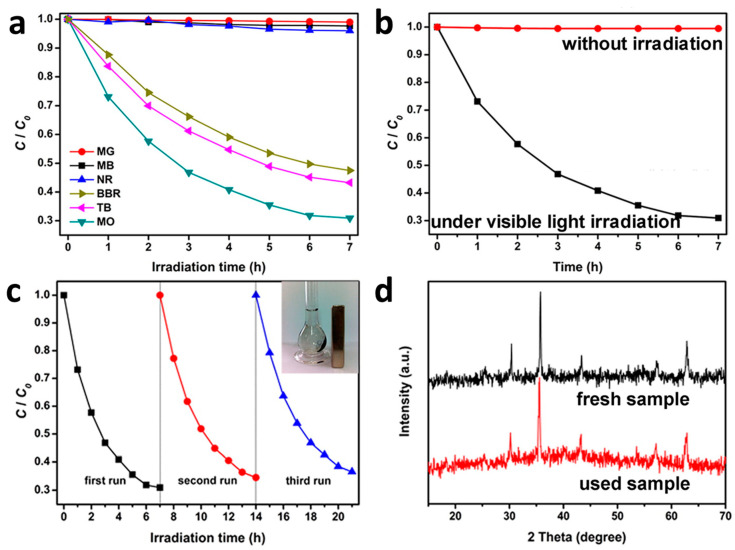
(**a**) Photodegradation of various dyes on TiO_2_-CoFe_2_O_4_-PANI. (**b**) Photodegradation of MO on TiO_2_-CoFe_2_O_4_-PANI without irradiation and under visible-light irradiation. (**c**) Photodegradation of MO on TiO_2_-CoFe_2_O_4_-PANI over several cycles. Inset shows the magnetic separation after photodegradation. (**d**) XRD patterns of TiO_2_-CoFe_2_O_4_-PANI before and after photocatalysis [34].

Nowadays, repeated recovery and reusability of photocatalysts are of great significance for practical applications in the environmental field. Therefore, it is important to synthesize and develop multicomponent nanocomposites based on metal oxides and conducting polymers for better catalytic performances in adsorption and photodegradation of organic dyes [34,37,110,111]. Moreover, the incorporation of magnetic nanoparticles in the composite photocatalysts, especially spinel ferrites and iron oxides, enables collection and reuse via magnetic separation [84]. Taking this into consideration, Xiong et al. prepared a magnetically recyclable photocatalyst based on a ternary composite of TiO_2_-CoFe_2_O^4^-PANI for the degradation of various dyes [34]. The results show that the ternary composite photocatalyst exhibits a high photocatalytic activity in the degradation of anionic dyes, such as MO, trypan blue (TB), and Brilliant Blue R (BBR), while only poor activity for cationic dyes, such as MB, Malachite Green (MG), and Neutral Red (NR) (Figure 6a). The higher efficiency in the degradation of anionic dyes can be attributed to an electrostatic attraction between the negatively charged groups of anionic dyes and the positively charged backbone of PANI, which greatly promotes the degradation. Moreover, the ternary composite showed a high photodegradation property rather than adsorption, where the photobleaching of MO mainly come from the photodegradation process (Figure 6b). Due to the good magnetic properties of CoFe_2_O_4_, the ternary TiO_2_-CoFe_2_O_4_-PANI photocatalyst also enables collection by a simple magnet or an applied magnetic field (see inset of Figure 6c). The stability of photocatalysts is also one of the most important factors for their use in practical applications [24,112,113]. Ternary composites of metal oxides and CPs demonstrated a high stability. For example, TiO_2_-CoFe_2_O_4_-PANI photocatalysts still retained a high rate of photodegradation after three cycles (Figure 6c), as well as structural stability (Figure 6d).

In summary, the decomposition of organic pollutants, especially dyes, is an important consideration in the application of both binary and ternary composites of metal oxides and CPs. Current strategies are focused on the development of multicomponent composites possessing a high photocatalytic activity along with a perfect recyclability and reusability.

### 4.2. CO_2_ Reduction

Over the past century, CO_2_ (recognized as a major greenhouse gas) levels have been rapidly increasing because of human activities, which has caused a global warming problem [114]. Among the various strategies developed for reducing CO_2_ emissions, photocatalytic CO_2_ reduction using semiconductors is one of the most viable approaches [115,116,117]. CO_2_ photoreduction is defined as the process of using light irradiation-induced energy to convert CO_2_ to reduced C1 and C2 hydrocarbon compounds [118]. However, CO_2_ is a very stable molecule and cannot absorb in the sunlight spectrum, and thus the photoreduction process needs support from suitable photosensitizers [99]. These photosensitizers are semiconductors that can generate electron–hole pairs and their subsequent transfer to CO_2_ and a reductant, respectively. A wide range of metal oxides, such as TiO_2_, Ga_2_O_3_, W_18_O_49_, SrTiO_3_, ZnGa_2_O_4_, Zn_2_GeO_4_, and Bi_2_WO_6_, have been used as semiconductors for the photocatalytic reduction of CO_2_ with H_2_O [119,120,121,122]. Nonetheless, it has been indicated that their poor photocatalytic performance is caused by the rapid recombination of photogenerated electrons and holes, low CO_2_ adsorption ability, and low CO_2_ reactivity.

**Figure 7:**
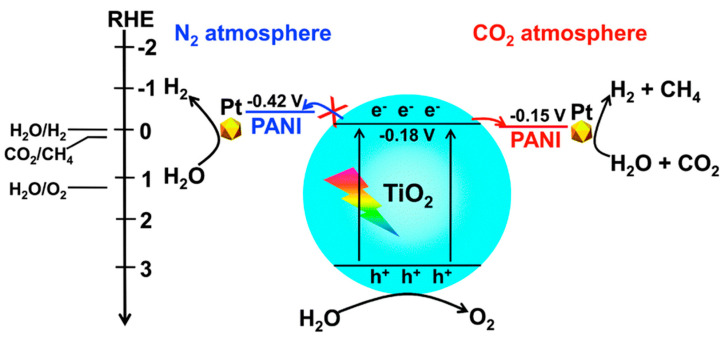
Schematic illustration of the mechanism for CO_2_ photoreduction using a binary PANI/TiO_2_ composite [99].

It has to be noted that the application of using conducting polymers as a single photocatalyst for CO_2_ photoreduction is relatively limited mainly due to their low photostability. Therefore, conducting polymers, such as PANI, PPy, and PEDOT, have recently been combined with metal oxides in binary or ternary composites for CO_2_ photo-reducing applications. Liu et al. reported a PANI/TiO_2_ composite photocatalyst that demonstrated a considerable enhancement in the photoreduction of CO_2_ with H_2_O [118]. This enhancement was ascribed to an increase in CO_2_ chemisorption and the facilitated separation of photogenerated electron–hole pairs. The specific mechanism for CO_2_ photoreduction is presented in Figure 7. It has been indicated that the LUMO level or CB edge of TiO_2_ has the same level of 0.18 V under N_2_ and CO_2_ conditions. However, the LUMO level of PANI is different under N_2_ and CO_2_ conditions, at −0.42 V and −0.15 V, respectively. Consequently, electrons were transferred from the CB of TiO_2_ to PANI under a CO_2_ atmosphere due to the change in the LUMO level of PANI. It was proposed that this phenomenon makes a large contribution to the improvement in the separation of photogenerated electron–hole pairs in the binary PANI/TiO_2_ composite, which leads to a higher performance in the photoreduction of CO_2_ and H_2_O to CH_4_ and H_2_. In particular, the binary composite showed higher rates of CO, CH_4_, and H_2_ formation (2.8, 3.8, and 2.7 times, respectively) from CO_2_ photoreduction, as compared with TiO_2_. It was also suggested that the synergistic effect between TiO_2_ and PANI largely reduced CO_2_ in the presence of H_2_O. This work may be regarded as the first related to the use of binary conducting polymer/metal oxide composite for CO_2_ photoreduction. In another study, Gao et al. used another conducting polymer (PPy) in combination with TiO_2_ in a binary PPy/TiO_2_ nanocomposite for the reduction of CO_2_ [32]. The composite was synthesized using simple oxidative polymerization of pyrrole using ferric chloride (FeCl_3_) as the oxidant in the presence of TiO_2_ nanoparticles. The nanocomposite showed better photoreduction efficiency for CO_2_ than pure TiO_2_ under simulated solar light irradiation. In addition, the PPy/TiO_2_ photocatalyst has high potential for practical applications because of its high stability. In summary, these studies indicate that the composites of conducting polymers and metal oxides are potential candidates for the development of advanced materials for the reduction of CO_2_.

### 4.3. Photocatalytic Oxidation of Heavy Metals

**Figure 8:**
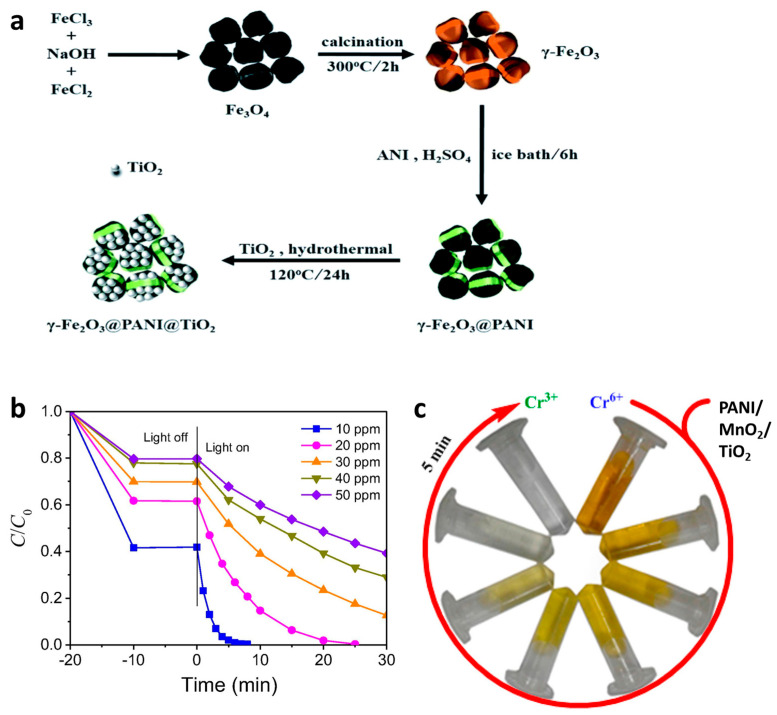
(**a**) Schematic illustration of the preparation procedure for ternary γ-Fe_2_O_3_/PANI/TiO_2_ composite for As(III) photoreduction [131]. (**b**) Liquid-phase photocatalytic Cr(VI) anion reduction by the binary PANI/TiO_2_ composite [100]. (**c**) Photographic images of Cr(VI) reduction by ternary PANI/MnO_2_/TiO_2_ nanocomposite [132].

Arsenic (As), is one of the heavy metals present in water environments, and it can pose a serious threat to humans and other species because of its high toxicity [123,124]. Generally, As(III) (arsenite) and As(V) (arsenate) are the two most common types of As present in groundwater, of which As(III) shows a higher toxicity. Current removal technologies exhibit poor performance with regard to As(III), due to its very high mobility [125]. It has been proposed that one of the most efficient approaches for removal is the conversion of As(III) to As(V), which then enables the removal of As(V) by an adsorption process [126]. Recently, photocatalyst-based oxidation methods have attracted great attention for the removal of heavy metals [127,128]. Therefore, there has been increasing attention devoted to the development of novel materials for an efficient oxidation of As(III) to As(V) and simultaneous removal of As(V). Due to an effective adsorption of As(V) onto the surface, good magnetic properties, and easy separation, γ-Fe_2_O_3_ has recently been used in fabricating photocatalysts for As(III) removal, in combination with TiO_2_ [129,130]. Taking advantage of the synergistic effects of TiO_2_ and γ-Fe_2_O_3_ along with the support of PANI, Wang et al. successfully developed a novel ternary composite (γ-Fe_2_O_3_/PANI/TiO_2_) and significantly enhanced the photocatalytic adsorption of As(III) [131]. Regarding the preparation process, γ-Fe_2_O_3_ was first synthesized by annealing Fe_3_O_4_ at 300 °C for 2 h under air atmosphere (Figure 8a). A magnetic γ-Fe_2_O_3_ core–shell ternary nanocomposite was then obtained by hydrothermal crystallization of TiO_2_ on the surface of a magnetic core–shell loaded with PANI. The results indicate that the γ-Fe_2_O_3_/PANI/TiO_2_ composite effectively removed aqueous As(III) via a coupled photocatalytic oxidation/adsorption process. Specifically, the As(V) concentration in the solution increased and accounted for 54% of the total arsenic after 300 min of treatment with the γ-Fe_2_O_3_/PANI/TiO_2_ composite under visible-light irradiation. The adsorption process of As(III) was almost balanced, and its photocatalytic oxidation efficiency reached 75%. Notably, the photocatalytic oxidation of As (III) is affected by the synergic effects of some active substances, especially superoxide free radicals and photogenerated holes. The nanocomposite also showed excellent stability and good reusability.

Chromium (Cr) is another heavy metal that has a high toxicity to human beings. Cr can exist in various valance states but occurs mainly as Cr(VI) and Cr(III) [133]. Cr(VI) has a much higher toxicity for humans than Cr(III) [134]. Accordingly, it is necessary to design and develop advanced materials which can convert Cr(VI) into Cr(III). Recently, the photocatalytic reduction method has been considered as a promising approach for reducing toxic Cr(VI) to nontoxic Cr(III), due to its efficiency, simplicity, low cost, and convenience [45,135,136]. It has been demonstrated that photoreduction efficiency largely depends on the adsorption and diffusion of Cr(VI) ions on the surface of the photocatalyst. Due to its high adsorption ability, it has been suggested that PANI is a material that shows good potential for facilitating the photocatalytic reduction process of Cr(VI) through incorporation with other photocatalysts [137,138]. Taking this into consideration, Deng et al. developed a binary PANI/TiO_2_ composite for improving the photocatalytic reduction performance and stability of Cr(VI) ions [100]. This enhancement was due to two reasons: (i) PANI possesses many positively charged amino groups that enable the effective adsorption of Cr(VI) but make Cr(III) leave the reaction interface quickly, resulting in a high promotion of the photocatalytic reduction process and improvement in the photocatalyst stability; and (ii) modification of the TiO_2_ surface by PANI promotes the separation of photogenic charges on the TiO_2_ surface, leading to a great increase in photocatalytic activity. Specifically, the photocatalytic reduction performance of a binary PANI/TiO_2_ composite for different concentrations of Cr(VI) is presented in Figure 8b. Photocatalytic reduction is inversely proportional to the increase in concentration of Cr(VI), and Cr(VI) was completely removed to concentrations below 20 ppm after 30 min of light irradiation. It is difficult to separate the reactant product Cr(III) from the catalyst surface for a high concentration of Cr(VI), resulting in a covered photoreduction active site and reduced photocatalytic activity.

Moreover, the binary PANI/TiO_2_ composite showed a high stability while retaining 100% of the reduction performance after ten cycles. In order to further enhance the photocatalytic reduction and adsorption of binary PANI/TiO_2_ composites for high concentrations of Cr(VI), Vellaichamy et al. synthesized a ternary PANI/MnO_2_/TiO_2_ nanocomposite via a one-pot oxidative polymerization method at room temperature [132]. In this composite, TiO_2_ plays an important role as efficient linker between PANI and MnO_2_ [139,140]. Due to the synergistic effects between the three components, the ternary PANI/MnO_2_/TiO_2_ nanocomposite showed a superior photocatalytic activity in the reduction of toxic Cr(VI) to benign Cr(III) in comparison with single photocatalysts (PANI, MnO_2_, and TiO_2_) and binary composites (PANI/MnO_2_, PANI/TiO_2_, and MnO_2_/TiO_2_) (Figure 8c). Especially, PANI/MnO_2_/TiO_2_ revealed an excellent photocatalytic performance in the reduction of Cr(VI), with a transformation efficiency of 99.9% within 5 min and a rate constant of 15.97 × 10^−2^ min^−1^. The reduction rate was found to depend on the initial Cr(VI) concentration, oxidant (HCOOH), pH, and temperature. In addition, the PANI/MnO_2_/TiO_2_ nanocomposite also displayed good stability and retained a high photocatalytic efficiency, even after use in five cycles.

In summary, the results of these studies demonstrate that both binary and ternary composites of conducting polymers and metal oxides can be used as effective and economically viable photocatalysts for the reduction of toxic heavy metals in water.

## 5. Conclusions

The development of highly active composite photocatalysts for use in environmental applications is considered to be a sustainable way of eliminating organic pollutants and heavy metals. Among them, composite photocatalysts based on inorganic semiconductors, especially metal oxides, and CPs emerge as novel promising photoactive materials. It was demonstrated that these composite photocatalysts have several outstanding characteristics, such as light absorption in the visible range of the spectrum, high photocatalytic activity and stability, good reusability, low cost, convenience, and scalability of production. Over the past few decades, numerous composite photocatalysts based on conducting polymers and metal oxides were prepared and developed. These can be classified into two types based on the number of components in the composites, including (i) binary composite photocatalysts involving the use of one conducting polymer (i.e., PANI, PEDOT, and PPy) and one metal oxide (i.e., TiO_2_, Fe_3_O_4_, SnO_2_, and ZnO); and (ii) ternary composite photocatalysts, which often include one conducting polymer and two different metal oxides or one conducting polymer, one metal oxide, and another semiconductor. Both binary and ternary conducting polymer/metal oxide composites show promising photocatalytic activity for the degradation, reduction, and adsorption of organic pollutants, CO_2_ gas, and heavy metals. However, ternary composites were confirmed as superior to binary ones due to the synergistic effects of three components in promoting photocatalytic activity and improving photocatalyst stability. Therefore, conducting polymer/metal oxide composites are currently one of the most promising candidates for photocatalysts in environmental applications.

## Figures and Tables

**Table 1:** Summarized properties and applications of conducting polymer/metal oxide composites as photocatalysts.

Composite	Band-Gap Energy (eV)	Photocatalytic Properties	Applications	Reference
**Binary Composite of CPs and Metal Oxides**				
Mesoporous PANI/TiO_2_	-	Enhanced water oxidation efficiency under sunlight irradiation, reaching about two-fold higher photocurrent densities than pure TiO_2_ nanoparticles	Water splitting	[20]
PANI/TiO_2_ nanorods	3.1	- Quantum yield: 9.86 × 10^−5^ molecules/photon and 2.82 × 10^−5^ molecules/photon for PANI/TiO_2_ and TiO_2_, respectively. - PANI/TiO_2_ showed better performance than TiO_2_ with a rate constant of 4.46 × 10^−2^ min^−1^ compared with 2.18 × 10^−2^ min^−1^, respectively	Degradation of organic pollutants (Bisphenol A)	[21]
PANI nanobelt/TiO_2_	2.77	The photocatalytic degradation rate of rhodamine B was 99%	Degradation of rhodamine B	[22]
PANI/TiO_2_ nanotubes	-	The photocatalytic activity can easily be tuned using a particular type and concentration of the acid dopant in the redoping process	Degradation of rhodamine B	[23]
PANI/ZnO	-	- The composite exhibits a dramatic photocatalytic activity both under ultraviolet and visible-light irradiation - The photo-corrosion of ZnO was successfully inhibited	Degradation of methylene blue (MB)	[24]
PANI/ZnO	2.13–2.22	The composite photocatalysts’ activity was broadened into the Vis region	Degradation of acid blue	[25]
PANI/Fe_3_O_4_	-	The adsorption process prevails in relation to photodegradation	Degradation of MB	[26]
PANI/SnO_2_	2.7	Increased photocatalytic activity for visible light is due to its electrical conductivity and efficient charge separation	Degradation of direct blue 15	[27]
PANI/Sn_3_O_4_	2.06	Photocatalytic activity for visible light is 2.27 times higher than that of Sn_3_O_4_ alone	Degradation of rhodamine B	[28]
PANI/CoFe_2_O_4_	-	Photocatalytic activity under visible-light irradiation with CoFe_2_O_4_/PANI was 80 times greater than for CoFe_2_O_4_	Degradation of methyl orange (MO)	[29]
PEDOT/TiO_2_	3.01–3.05	PEDOT infused TiO_2_ nanofiber, exhibits the highest degradation enhancement (125%)	Degradation of phenazopyridine	[30]
PPy/TiO_2_	-	The photoactivity of the nanocomposite arises from the electron transfer from excited PPy to TiO_2_ nanoparticles and further across the nanocomposite interface	Degradation of MB	[31]
PPy/TiO_2_	3.08–3.11	The photoactivity of nanocomposites increased by 41% compared with pure TiO_2_	Degradation of RhB and CO_2_	[32]
ZnO-microrods/PPy	1.7	Composite films achieve a much higher photocatalytic efficiency in comparison with pure ZnO-microrod arrays (a rate of 22%/min MB degradation)	Degradation of MB	[33]
**Ternary Composites**				
TiO_2_-CoFe_2_O_4_-PANI	-	The ternary TiO_2_-CoFe_2_O_4_-PANI composite shows a highly enhanced photocatalytic activity in the range of visible light, compared with the binary TiO_2_-CoFe_2_O_4_, CoFe_2_O_4_-PANI, or TiO_2_-PANI composites	Degradation of methyl orange	[34]
ZnFe_2_O_4_-TiO_2_-PANI	-	The decontaminating efficiency of composites on MO and RhB reached up to 98%	Degradation and adsorption of MO and RhB	[35]
rGO-ZnFe_2_O_4_-PANI	-	The photocatalytic activity still stays above 90% after five recycles	Degradation of RhB	[36]
ZnO/rGO/PANI	-	The photocatalyst shows an enhanced photocatalytic performance in the photodegradation of MO (almost 100%)	Degradation of methyl orange	[37]
Cu_2_O/ZnO-PANI	2.68	The ternary composite with Z-scheme heterojunction properties displayed outstanding adsorption properties, super-fast photocatalytic activities as well as enhanced stability	Degradation of congo red	[38]
PANI/TiO_2_/graphene	2.1	High photocatalytic activity is partly due to the sensitizing effect of PANI and the low recombination rate due to the graphene electron scavenging property	Degradation of MB	[39]
PANI-rGO-MnO_2_	1.92	The ternary composite exhibited significantly enhanced catalytic and photocatalytic activity under visible-light irradiation within 2 h	Degradation of MB	[40]
g-C_3_N_4_/TiO_2_/PANI	2.58	Greatly enhanced photocatalytic degradation and high reusability	Degradation of congo red	[41]

## Data Availability

Not applicable.
